# Prevalence of anastomotic leak and the impact of indocyanine green fluorescein imaging for evaluating blood flow in the gastric conduit following esophageal cancer surgery

**DOI:** 10.1007/s10388-017-0585-5

**Published:** 2017-06-28

**Authors:** Masaki Ohi, Yuji Toiyama, Yasuhiko Mohri, Susumu Saigusa, Takashi Ichikawa, Tadanobu Shimura, Hiromi Yasuda, Yoshiki Okita, Shigeyuki Yoshiyama, Minako Kobayashi, Toshimitsu Araki, Yasuhiro Inoue, Masato Kusunoki

**Affiliations:** 1 Department of Gastrointestinal and Pediatric Surgery, Mie University Graduate School of Medicine, Edobashi 2-174, Tsu, Mie 514-8507 Japan; 2 Department of Innovative Surgery, Mie University Graduate School of Medicine, Edobashi 2-174, Tsu, Mie 514-8507 Japan

**Keywords:** Esophagogastric anastomosis, Indocyanine green fluorescein imaging, Anastomotic leak

## Abstract

**Backgrounds and aim:**

Anastomotic leak (AL) following esophagectomy for esophageal cancer (EC) remains an important cause of prolonged hospitalization and impaired quality of life. Recently, indocyanine green (ICG) fluorescein imaging has been used to evaluate the gastric conduit blood supply during EC surgery. Although several factors have been reported to be associated with AL, no studies have evaluated the relationships between risk factors for AL, including ICG fluorescein imaging. The purpose of this study was to investigate the risk factors associated with AL following esophagectomy and to evaluate the impact of ICG fluorescein imaging of the gastric conduit during EC surgery.

**Methods:**

One hundred and twenty patients undergoing esophagectomy with esophagogastric anastomosis for EC were enrolled in this retrospective study. Clinicopathological factors, preoperative laboratory variables, and surgical factors, including ICG fluorescence imaging, were analyzed to determine their association with AL. Univariate and multivariate logistic regression analysis was used to evaluate the impact of each of these factors on the incidence of AL.

**Results:**

Among the 120 patients enrolled in the study, 10 (8.3%) developed AL. Univariate analysis demonstrated an increased risk of AL in patients with a high-neutrophil-to-lymphocyte ratio (*p* = 0.0500) and in patients who did not undergo ICG fluorescein imaging (*p* = 0.0057). Multivariate analysis revealed that the absence of ICG imaging was an independent risk factor for AL (*p* = 0.0098).

**Conclusions:**

Using ICG fluorescein imaging to evaluate blood flow in the gastric conduit might decrease the incidence of AL following EC surgery.

## Introduction

Death due to esophageal cancer (EC) is the sixth most common form of cancer-related mortality worldwide due to the high malignant potential of EC and its poor prognosis [1]. Although esophagectomy with lymphadenectomy is a highly invasive surgical procedure, postoperative outcomes have been improved with recent advancements in surgical technique and perioperative management [2]. However, anastomotic leak (AL) following esophagectomy remains an important source of in-hospital morbidity and mortality. AL is associated with a range of complications, including mediastinitis, sepsis, acute respiratory distress syndrome, and death [3, 4], as well as prolonged hospitalization, increased costs of medical treatment, and decreased quality of life. Furthermore, AL negatively impacts long-term survival [5]. The reported prevalence of AL ranges from 5 to 25% [6–8]. In a recent report based on a nationwide Japanese web-based database, AL was observed in 711 of 5354 patients undergoing esophagectomy (13.3%) [9]. Although no precise consensus exists, various risk factors for AL following esophagectomy have been identified [8, 10, 11]. Poor blood supply to the proximal part of the gastric conduit is one of the most important risk factors associated with postoperative AL following EC surgery [7, 12, 13]. Hence, techniques that identify regions of adequate and inadequate blood supply may help reduce the risk of AL following esophagectomy.

Indocyanine green (ICG) has long been used to evaluate liver function. After injection into the blood stream, ICG is distributed throughout the circulatory system and can be fluorescently visualized within tissues. The fluorescence intensity can be used to evaluate the vascular supply of tissues. Recently, ICG fluorescein imaging has been used to visualize the blood supply after anastomosis during vascular surgery, and to detect sentinel lymph nodes in breast cancer, gastric cancer, and colorectal cancer surgery [14–16]. In recent reports, the method has been shown to be an accurate tool for assessing microperfusion of gastrointestinal anastomoses and has been associated with improved anastomotic healing following colorectal surgery [17]. Similar results might be expected following EC surgery, in which the method can be used to evaluate blood flow in the gastric conduit.

The aim of this study was to investigate the risk factors associated with AL following esophagectomy and to evaluate the impact of ICG fluorescein imaging of the gastric conduit during EC surgery.

## Methods

### Patients

Between January 2000 and December 2015, 133 patients underwent esophagectomy with lymphadenectomy for EC at the Department of Gastrointestinal and Pediatric Surgery of Mie University Graduate School of Medicine. Among the 133 patients, 13 patients underwent two-stage surgery or reconstruction with colonic or jejunal tissue. Hence, a total of 120 patients who underwent esophagectomy with esophagogastric anastomosis for EC were enrolled in the study. Patient characteristics were collected, including demographic data [sex, age, body mass index (BMI), and American Society of Anesthesiologists (ASA) classification], tumor-specific data (T classification, lymph node metastasis, pathological type, and lymphatic and venous invasion), preoperative biochemical variables (tumor markers and systematic inflammatory indicators), and surgery-related factors (estimated blood loss, duration of surgery, surgical approach, anastomosis location, route of reconstruction, and method of anastomosis). These factors were evaluated, along with ICG fluorescein imaging, to identify risk factors associated with AL. Univariate and multivariate analyses were performed to detect the demographic, tumor specific, laboratory, and surgical factors affecting AL. Peripheral blood samples were collected from patients prior to surgery and included neutrophil and lymphocyte counts, albumin (Alb) level, and C-reactive protein (CRP) level. The neutrophil and lymphocyte counts were used to calculate the neutrophil-to-lymphocyte ratio (NLR), and the CRP level was used to calculate the modified Glasgow prognosis score (mGPS). The cut-off values for carcinoembryonic antigen (CEA) and squamous cell carcinoma antigen (SCC) were 5 and 1.5 ng/ml, respectively, according to the normal range used at our institution. The cut-off values for Alb and CRP were 3.5 g/dl and 0.5 mg/dl, respectively, based on reference values of the mGPS [18]. The mGPS was designed to assess the nutritional status and prognosis of patients undergoing surgery for gastrointestinal malignancies, and patients were categorized according to a score of 0, 1, or 2, based on Alb and CRP values. NLR was designed as an index of systematic inflammation and its prognostic value has been studied in many types of cancer. Patients were divided into two groups using an NLR cut-off value of 2.5. Finally, patients were divided into two groups based on BMI, based on a cut-off value less than the 25th percentile (19.4 kg/m^2^).

### Forming the gastric conduit

A gastric conduit 4 cm wide was made by stapling the lesser curvature of the stomach. The right gastric artery, right gastroepiploic artery, and branches of the left gastroepiploic arteries were preserved and provided the vascular supply to the gastric conduit through an arcade of peripheral vessels. The omentum was freed from the transverse colon and divided at the edge adherent to the colon so vessel communications and a sufficient amount of omentum were fully preserved.

### Evaluation of ICG fluorescein imaging and esophagogastric anastomoses

**Fig. 1:**
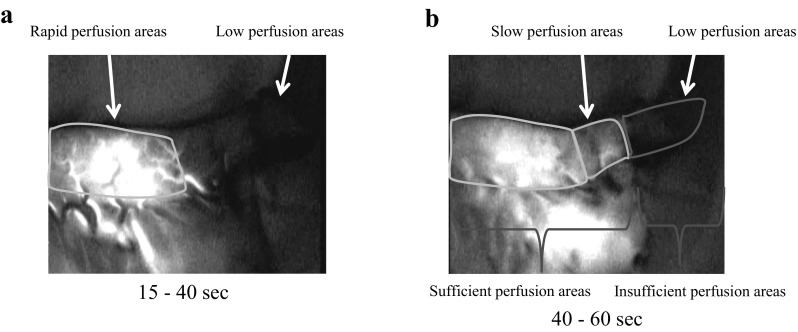
Intraoperative view of ICG fluorescence imaging of the gastric conduit. **a** Regions that were visualized between 15 and 40 s after ICG injection were categorized as rapid perfusion areas (*left side arrow*), and regions that were not visualized after injection were categorized as low perfusion areas (*right side arrow*). **b** Regions that were visualized between 40 and 60 s after ICG injection were categorized as slow perfusion areas (*left side arrow*), and regions that were not visualized were categorized as low perfusion areas (*right side arrow*). Vascular territories with rapid, slow, and low perfusion areas were identified about 15–60 s after injection. We defined rapid or slow perfusion areas as sufficient perfusion areas and low perfusion areas as insufficient perfusion areas

ICG fluorescein imaging was performed as follows: a 2.5 mg bolus injection of ICG dye (Diagnogreen; Daiichi-Sankyo Pharmaceutical, Tokyo, Japan) was administered after forming the gastric conduit. Vascular networks were assessed within the gastric wall and omentum about 15–60 s after ICG injection using an infrared ray imaging system (PDE; Hamamatsu Photonics K.K, Hamamatsu, Japan), and the data are recorded as a movie file [14]. Real-time visualization of the tissue perfusion enabled the operating surgeon to immediately calculate the perfusion index in the regions of interest using specially designed software (ROIs; Hamamatsu Photonics K.K, Hamamatsu, Japan), as detailed previously [19]. Based on the blood flow evaluation of the gastric conduit by ICG fluorescein imaging, the vascular territories with rapid, slow, and low perfusion areas were identified, and the border between each regions was marked (Fig. 1a, b). We defined rapid or slow (sufficient) perfusion areas as safely anastomosed, and low (insufficient) perfusion areas as unsafe for anastomosis [19] (Fig. 1b). In cases of sufficient perfusion at the level of the superior border of the sternum, cervical esophagogastric end-to-side anastomoses were generally performed using a circular stapler via a retrosternal route. This is one of the most common reconstruction methods in Japan, as reported in a comprehensive registry of esophageal cancer patients [20]. On the other hand, when blood flow to the gastric conduit was deemed insufficient for end-to-side anastomoses at the level of the superior border of the sternum, we constructed end-to-end anastomoses using a hand-sewn technique, adding vessels anastomosed between the short gastric vein and artery in the neck vessels. Alternatively, we used an adjunctive procedure whereby we decided good blood flow site of gastric conduit via sharpening part of the manubrium or by making a longitudinal incision over the manubrium and upper part of the sternum to make a secure anastomosis.

Cases that were not evaluated by ICG fluorescein imaging were included in the ICG (−) group, and cases that were evaluated by ICG fluorescein imaging were included in the ICG (+) group.

### Definition of postoperative complications

AL was defined as any esophagogastric anastomosis dehiscence that was clinically symptomatic (abscess, mediastinitis, externalized drainage of digestive fluid) or clinically asymptomatic but detected by contrast study within 30 days after esophagectomy, and included necrosis of the gastric conduit and anastomotic-bronchial fistulas. AL was assessed by a water-soluble, monomeric, ionic X-ray contrast medium (Gastrografin; Schering AG, Berlin, Germany) on postoperative day 6. In cases of uncertainty, the diagnosis was confirmed by an upper gastrointestinal endoscopy. Surgical site infections was defined as superficial pus expressed from the abdominal, thoracic, or drains incision sites, requiring surgical debridement and antibiotic treatment. Respiratory complications included bronchial circulatory disturbance, disorders of ventilation, atelectasis, pneumonia, respiratory failure, and acute respiratory distress syndrome. Recurrent laryngeal nerve paralysis was defined as a disturbance of vocal cord mobility with insufficient glottics closure as observed with flexible laryngoscopy, and was recorded according to the affected side.

### Statistical analysis

The data of continuous variables such as age, estimated blood loss and duration of operation were presented as median with 25th percentile and 75th percentile. Comparisons were made using the Mann–Whitney or Kruskal–Wallis tests, as appropriate. Correlations were analyzed by Spearman’s coefficient analysis. Univariate and multivariate analyses were performed using logistic regression analyses to determine the risk factors affecting AL. Parameters with *p* < 0.1 in univariate analyses were considered statistically significant and were used in the multivariate analysis. All statistical analyses were carried out using JMP software, version 10 (SAS Institute, Cary, NC, USA).

## Results

### Patients and surgical characteristics

The study group was comprised of 101 males and 19 females and the median age was 68 years old (25th percentile; 63 and 75th percentile; 74). Characteristics of the study population are shown in Table 1. According to the Japanese classification of EC (10th Ed.) [21], pathological stages included stage 0 (14 patients, 11.7%), stage I (31 patients, 25.8%), stage II (40 patients, 33.3%), stage III (24 patients, 20%), and stage VIa (11 patients, 9.2%). Fifty-nine (49.2%) patients underwent ICG fluorescein imaging to evaluate blood flow in the gastric conduit. Median estimated blood loss was 495.0 g (25th percentile; 305.5 g and 75th percentile; 719.8 g) and median duration of operation was 551.0 min (25th percentile; 420.5 min and 75th percentile; 632.0 min).

**Table 1:** Characteristic of the study population (*n* = 120)

Variables	Number	Median (25th percentile, 75th percentile)
Gender		
Male	101 (84.2%)	
Female	19 (15.8%)	
Age (years)		68 (63, 74)
ASA classification		
1	55 (45.8%)	
≥2	65 (54.2%)	
Tumor location		
Upper	11 (9.2%)	
Middle	55 (45.8%)	
Lower	54 (45%)	
Pathological T		
T0, T1a, T1b, T2	80 (66.7%)	
T3, T4	40 (33.3%)	
Pathological N		
Absent	64 (53.3%)	
Present	56 (46.7%)	
Lymphatic invasion		
Absent	57 (47.5%)	
Present	63 (52.5%)	
Venous invasion		
Absent	81 (67.5%)	
Present	39 (32.5%)	
Pathology		
SCC	108 (90.0%)	
Others	12 (10.0%)	
Pathological stage		
0	14 (11.7%)	
I	31 (25.8%)	
II	40 (33.3%)	
III	24 (20.0%)	
IV	11 (9.2%)	
Preoperative treatment		
Absent	64 (53.3%)	
Present	56 (46.7%)	
Estimated blood loss (g)		495.0 (305.5, 719.8)
Duration of operation (min)		551.0 (420.5, 632.0)
Approach of operation		
Open	33 (27.5%)	
Thoracoscopic	87 (72.5%)	
Lymphadenectomy		
Two-field	53 (44.2%)	
Three-field	67 (55.8%)	
Location of anastomosis		
Cervical	73 (60.8%)	
Intrathoracic	47 (39.2%)	
Route of reconstruction		
Subcutaneous	4 (3.3%)	
Retrosternal	61 (50.8%)	
Posterior mediastinal	55 (45.8%)	
Method of anastomosis		
End-to-side	112 (93.3%)	
End-to-end	8 (6.7%)	
ICG fluorescein imaging		
ICG (−) group	61 (50.8%)	
ICG (+) group	59 (49.2%)	
Complications		
Anastomotic leakage	8 (6.7%)	
Necrosis of gastric conduit	1 (0.83%)	
Anastomotic-bronchial fistula	1 (0.83%)	
Surgical site infection	24 (20.0%)	
Respiratory complication	23 (19.2%)	
Recurrent laryngeal nerve paralysis	14 (11.7%)	

*ASA* American society of anesthesiologists, *SCC* squamous cell carcinoma, *ICG* indocyanine green

Among the 120 study patients, 10 (8.3%) developed AL. Of those 10 patients, 1 patient (0.83%) developed necrosis of the gastric conduit and another patient (0.83%) developed an anastomotic-bronchial fistula. In addition, a total of 24 (20.0%), 23 (19.2%), and 14 (11.7%) patients had surgical site infections, respiratory complications, or recurrent laryngeal nerve paralysis, respectively (Table 1).

### Association between various factors and postoperative AL

A univariate analysis showed no significant associations between AL and demographic data (age, sex, comorbidities, preoperative treatment, and BMI). Furthermore, no significant associations between AL and clinicopathological factors were observed (Table 2). Preoperative tumor markers, Alb, CRP, and mGPS were not associated with the development of AL, but NLR tended to be higher in patients who developed AL [HR 3.8130, 95% confidence interval (CI) 0.9999–18.4563, *p* = 0.0500] (Table 2). No significant associations between AL and surgery-related factors were observed (estimated blood loss, duration of surgery, surgical approach, anastomosis location, reconstruction route, and method of anastomosis). However, the ICG (−) group had a significantly higher incidence of postoperative AL compared with the ICG (+) group [HR 10.0384, 95% confidence interval (CI) 1.7969–188.2576, *p* = 0.0057] (Table 2).

**Table 2:** Various factors and postoperative AL in patients undergoing esophagectomy: univariate logistic regression analysis

Variables	AL (−)	AL (+)	HR	95% CI	*p* value
Gender					
Male	93 (92.1%)	8 (7.9%)	0.7312	0.1650–5.1191	0.7143
Female	17 (89.5%)	2 (10.5%)			
Age (years)					
>68	51 (91.1%)	5 (8.9%)	1.8000	0.4873–7.3726	0.3773
≤68	60 (93.8%)	4 (6.2%)			
BMI (kg/m^2^)					
≥19.4	83 (90.2%)	9 (9.8%)	3.0731	0.5412–57.9333	0.2340
<19.4	28 (96.6%)	1 (3.4%)			
ASA classification					
≥2	58 (89.2%)	7 (10.8%)	2.0920	0.5505–10.0919	0.5788
1	52 (94.5%)	3 (5.5%)			
Preoperative therapy					
Present	50 (89.3%)	6 (10.7%)	1.8000	0.4873–7.3726	0.3773
Absent	60 (93.8%)	4 (6.2%)			
T classification					
T3, T4	36 (90%)	4 (10%)	1.3704	0.3329–5.1011	0.6448
T0, T1a, T1b, T2	72 (92.3%)	6 (7.7%)			
Lymphatic node metastasis					
Present	49 (87.5%)	7 (12.5%)	2.9048	0.7643–14.0202	0.1195
Absent	61 (95.3%)	3 (4.7%)			
Pathology					
Others	12 (100%)	0 (0%)	0.00013	0.0000–1.8782	0.1375
SCC	98 (90.7%)	10 (9.3%)			
Lymphatic invasion					
Present	56 (88.9%)	7 (11.1%)	3.3124	0.7602–22.9017	0.1152
Absent	53 (96.4%)	2 (3.6%)			
Venous invasion					
Present	37 (92.5%)	2 (7.5%)	0.5560	0.0802–2.4384	0.4582
Absent	72 (91.1%)	7 (8.9%)			
CEA (ng/µl)					
>5	27 (90%)	3 (10%)	1.3148	0.2631–5.3642	0.7160
≤5	71 (92.2%)	6 (7.8%)			
SCC (ng/dl)					
>1.5	20 (83.3%)	4 (16.7%)	2.8333	0.6722–10.8862	0.1473
≤1.5	85 (93.4%)	6 (6.6%)			
Alb (g/dl)					
<3.5	11 (91.7%)	1 (8.3%)	1.0000	0.0518–6.1171	1.0000
≥3.5	99 (91.7%)	9 (8.3%)			
CRP ( mg/dl)					
>0.5	26 (86.7%)	4 (13.3%)	2.1538	0.5178–8.1310	0.2748
≤0.5	84 (93.3%)	6 (6.7%)			
mGPS					
1–2	31 (88.6%)	4 (11.4%)	1.6989	0.4110–6.3597	0.4436
0	79 (92.9%)	6 (7.1%)			
NLR					
>2.5	41 (85.4%)	7 (14.6%)	3.8130	0.9999–18.4563	0.0500
≤2.5	67 (95.7%)	3 (4.3%)			
Estimated blood loss (ml)					
>495	56 (93.3%)	4 (6.7%)	0.6429	0.1570–2.3740	0.5076
≤495	54 (90%)	6 (10%)			
Duration of operation (min)					
>551	52 (88.1%)	7 (11.9%)	2.6026	0.6851–12.5565	0.1637
≤551	58 (95.1%)	3 (4.9%)			
Approach of operation					
Thoracoscopic	80 (92.0%)	7 (8.0%)	0.8750	0.2269–4.2605	0.8545
Open	30 (90.9%)	3 (9.4%)			
Lymphadenectomy					
Three-field	60 (89.6%)	7 (10.4%)	1.9444	0.5115–9.3818	0.3378
Two-field	50 (94.3%)	3 (5.7%)			
Location of anastomosis					
Cervical	65 (89.0%)	8 (11.0%)	2.7692	0.6565–18.9160	0.1757
Intrathoracic	45 (95.7%)	2 (4.3%)			
Route					
Retrosternal	55 (91.7%)	5 (8.3%)	1.1919	0.2972–5.0011	0.8105
Posterior mediastinal	52 (92.9%)	4 (7.1%)			
Method of anastomosis					
End-to-side	103 (92.0%)	9 (8.0%)	0.6117	0.0928–12.0783	0.6777
End-to-end	8 (88.9%)	1 (11.1%)			
ICG fluorescein imaging					
ICG (−) group	52 (85.2%)	9 (14.7%)	10.0384	1.7969–188.2576	0.0057
ICG (+) group	58 (98.3%)	1 (1.7%)			

*AL* anastomotic leak, *HR* hazard ratio, *CI* confidence interval, *BMI* body mass index, *ASA* American society of anesthesiologists, *SCC* squamous cell carcinoma, *CEA* carcinoembryonic antigen, *SCC* squamous cell carcinoma antigen, *Alb* albumin, *CRP* C-reactive protein, *mGPS* modified Glasgow prognosis score, *NLR* neutrophil-to-lymphocyte ratio, *ICG* indocyanine green

### ICG (−) is an independent risk factor for postoperative AL

Using the significant factors identified by univariate analysis, a multivariate analysis was performed to identify factors independently associated with AL, as shown in Table 3. We found that the absence of ICG fluorescein imaging was an independent risk factor for AL following EC surgery [HR 9.0740, 95% confidence interval (CI) 1.5923–171.2574, *p* = 0.0098].

**Table 3:** Multivariate logistic regression analysis for postoperative AL in EC patients

Variables	Multivariate analysis		
	HR	95% CI	*p* value
NLR (>2.5 vs ≤2.5)	3.2911	0.8306–16.3605	0.0909
ICG fluorescein imaging [ICG (−) group vs ICG (+) group]	9.0740	1.5923–171.2574	0.0098

*AL* anastomotic leak, *HR* hazard ratio, *CI* confidence interval, *NLR* neutrophil-to-lymphocyte ratio, *ICG* indocyanine green

## Discussion

Esophagogastric AL remains a relatively common complication following esophagectomy. Previously cited risk factors for AL include the number of preoperative comorbidities, advanced pathologic stage, nutritional status, previous esophagogastric operations, neoadjuvant therapy, anastomotic location, and anastomotic technique [22]. In the current study, we, for the first time, demonstrated that the absence of ICG fluorescein imaging is an independent predictor of postoperative AL.

It has been reported that 20% of the gastric fundus relies on blood supply from within the gastric wall [23], while the remaining blood supply to the gastric conduit comes from the right gastroepiploic artery. The blood supply to the surgical anastomosis is primarily provided by the local micro-vascular network within the fundus ventriculi. The blood supply to the anastomotic region is often subjectively assessed via relatively weak parameters, such as active bleeding from the resection margin or palpable pulsation in the gastric conduit. However, these parameters lack predictive accuracy, making the assessment of the border between sufficiently and insufficiently perfused regions difficult. In this study, we used ICG fluorescein imaging to objectively evaluate tissue perfusion. ICG fluorescein imaging has previously been validated as a method for evaluating the blood supply of the gastric conduit. Rino et al. reported that ICG fluorescence imaging provided excellent visualization of vascular networks within the gastric wall and omentum in EC patients [24]. In this study, the blood supply route started in the greater omentum beside the splenic hilum, similar to the route that supplied the greater curvature and the gastric wall. In most patients, the splenic hiatal vessels provided the major blood supply to the anastomosis. It is possible that this blood supply was the source of the slow perfusion areas in our study.

According to recent reports, ICG fluorescence imaging contributes to a decreased incidence of AL following colorectal surgery [17]. Some reports have demonstrated a similar advantage of using this method to evaluate blood flow to the gastric conduit or esophagogastric anastomosis in EC surgery [24–27]. Nakashima et al. used the pedicled omentum flap as a surgical option, and evaluated blood flow by ICG fluorescence imaging around the anastomosis with good results [25]. Zehetner et al. assessed and quantified graft perfusion in 150 consecutive EC patients using laser-assisted angiography [27]. AL was detected in 16.7% patients and was significantly less likely when the anastomosis was placed in an area of good perfusion. The authors concluded that intraoperative real-time assessment of perfusion correlated with the risk of AL and confirmed the critical relationship between good perfusion and anastomotic healing. To our knowledge, ours is the first clinical study to demonstrate that ICG fluorescein imaging is associated with a decreased risk of AL following EC surgery.

**Fig. 2:**
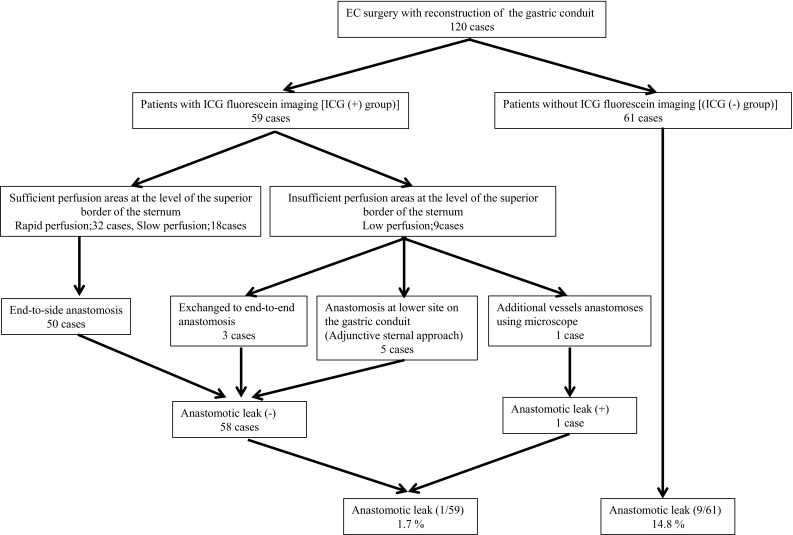
Clinical courses of the 59 patients who underwent ICG fluorescein imaging and the 61 patients who did not undergo ICG fluorescein imaging during EC surgery

In our study, 59 of 120 patients underwent evaluation of blood flow to the gastric conduit by ICG fluorescein imaging during EC surgery. Based on the results of ICG fluorescein imaging, 50 of these 59 patients underwent end-to-side anastomoses using a circular stapler in sufficiently perfused areas (rapid perfusion areas; 32 patients, slow perfusion areas; 18 patients) of the gastric conduit. Of the remaining 9 patients, 3 patients underwent end-to-end anastomosis at a region of the gastric conduit with sufficient perfusion. The remaining six patients had insufficient perfusion of the gastric conduit at the level of the superior border of the sternum. One patient required additional vessels anastomoses between the short gastric vein and artery in the neck vessels, and five patients required an adjunctive procedure in which a sternal approach was used to construct the anastomosis at a lower site on the gastric wall where there is better blood flow (Fig. 2). We observed only one case of AL in the 59 patients in whom ICG fluorescein imaging was used (1.7%). The patient underwent esophagogastric anastomosis with vessels anastomosed via the subcutaneous route. In contrast, 9 of the 61 patients who underwent anastomosis without ICG fluorescein imaging developed AL (14.8%). Hence, we were able to significantly reduce AL by using ICG fluorescein imaging.

Our study further demonstrated that preoperative high NLR has a high relationship with postoperative AL. NLR has been used to evaluate oncological outcomes such as early recurrence and poor prognosis. Recently, several researchers have examined NLR in the perioperative period, hypothesizing that an increased systemic inflammatory response can negatively impact surgical outcome in cancer patients. It has previously been shown that elevated NLR on postoperative day 1 is associated with postoperative complications [28]. In addition, the preoperative NLR value has been associated with an increased hospital length of stay [29]. Recently, we reported that high NLR was one of several independent variables associated with the development of postoperative infectious complications, including AL, following gastrectomy for gastric cancer [30]. These findings are consistent with our current results which show a high relationship between high NLR and AL. Hence, NLR might help to identify patients at increased risk for AL after esophagectomy.

This study has several limitations. First, this was an observational study based on medical record review. As such, our results depend upon adequate documentation of appropriate data. Second, our study population was relatively small. A larger study is required to validate our results.

In conclusion, we found that preoperative high NLR and the absence of ICG fluorescein imaging might be potent predictors of AL. The use of ICG fluorescein imaging to evaluate blood flow of the gastric conduit may contribute to a reduction in the incidence of AL following EC surgery.
